# Lack of an association of *miR-938 *SNP in IDDM10 with human type 1 diabetes

**DOI:** 10.1186/1758-5996-3-27

**Published:** 2011-10-20

**Authors:** Xiaofan Mi, Hongzhi He, Yangxin Deng, Abert M Levin, Jin-Xiong She, Qing-Sheng Mi, Li Zhou

**Affiliations:** 1 Henry Ford Immunology Program, Henry Ford Health System, 1 Ford Place, Detroit, MI 48202, USA; 2 Department of Dermatology, Henry Ford Health System, 1 Ford Place, Detroit, MI 48202, USA; 3 Division of Endocrinology, Diabetes, Bone and Mineral Disorders/Department of Internal Medicine, Henry Ford Health System, 1 Ford Place, Detroit, MI 48202, USA; 4 MedStart Program, Irvin D. Reid Honors College, Wayne State University, 5155 Gullen Mall, Detroit, MI 48202, USA; 5 Department of Endocrinology, Affiliated Hospital, Taishan Medical College, 706 Taishan Blvd, Taian, Shandong Province, 27100, P. R. China; 6 Department of Public Health Sciences, Henry Ford Health System, 1 Ford Place, Detroit, MI 48202, USA; 7 Center for Biotechnology and Genomic Medicine, Georgia Health Sciences University, 1120 15th street, Augusta, GA 30912, USA

**Keywords:** Type 1 diabetes, polymorphism, microRNAs

## Abstract

MicroRNAs (miRNAs) are a newly discovered type of small non-protein coding RNA that function in the inhibition of effective mRNA translation, and may serve as susceptibility genes for various disease developments. The SNP rs12416605, located in human type 1 diabetes IDDM10 locus, changes the seeding sequence (UGU[G/*A*]CCC) of miRNA *miR-938 *and potentially alters *miR-938 *targets, including IL-16 and IL-17A. In an attempt to test whether *miR-938 *may be a susceptibility gene for IDDM10, we assessed the possible association of the *miR-938 *SNP with T1D in an American Caucasian cohort of 622 patients and 723 healthy controls by TaqMan assay. Our current data do not support the association between the SNP in *miR-938 *and type 1 diabetes.

## To the Editor

In recent years, it has become evident that the traditional central dogma of DNA transcription into messenger RNA (mRNA) and mRNA translation into proteins describes only a part of the genetic information machinery that determines which proteins might possibly be synthesized. Additional genetic information is required to control the timing and rates of protein manufacturing processes. Although only a minority of the human genome (2-3%) codes for proteins, a large fraction of the non-protein coding genome is transcribed. MicroRNAs (miRNAs) are a newly discovered type of small non-protein coding RNAs (21-25nt) that function in the inhibition of effective mRNA translation through imperfectly base pairing with the 3' untranslated region (3'-UTR) of target mRNAs. miRNA targets are largely unknown but they are thought to target about 30%-80% of total human proteins at a frequency of about one to hundreds of target genes for a given miRNA. Previous studies indicate that miRNAs play a very important role in determining cellular development, apoptosis, differentiation, and proliferation. Recent studies from our group and others have reported that miRNAs are involved in immune cell development, including CD4^+^CD25^+^FoxP3^+ ^regulatory T cells and natural killer T cells. Furthermore, mice without expression of miRNAs in the bone marrow and the thymus develop autoimmune diseases [1,2]. Most importantly, miRNAs as susceptibility genes contribute to various disease developments, including cancer [3]. Human type 1 diabetes (T1D), or insulin-dependent diabetes mellitus (IDDM), is clearly a chronic autoimmune disorder caused by the interaction of environmental factors with an inherited predisposition. To date, about 50 IDDM susceptibility loci have been mapped to specific chromosome regions in the human. A long search for the gene(s) responsible for diabetes susceptibility has identified only very few candidate genes in past decades. This raises the possibility that the candidate search is a much more complex process than initially assumed, or that some non-coding regulatory genes were missed in previous studies. Given miRNA's suppressive nature and critical role in regulation, the possibility that miRNAs are T1D-risk genes may underlie the diverse findings of genetic studies, including evidence that protein altering gene polymorphisms are not generally found in T1D. Mutations located in critical areas of the mature miRNA potentially affect its structure or expression level, which may lead to abnormal protein-coded gene expression that is phenotypically similar to the disruption of the protein-coded gene itself. Thus, inadequate or miss-timed expression of a functional protein may occur either due to disruption of the DNA coding sequence, leading to a dysfunctional protein, or due to abnormal miRNA regulation of a normal gene. To date there is no direct evidence linking miRNAs and human T1D susceptibility. We previously mapped 27 miRNAs located in 9 T1D susceptibility regions [4], which places miRNAs as candidates for T1D susceptibility genes. Here, using available single nucleotide polymorphism (SNP) data bases, we found the SNP rs12416605 in IDDM10 is located in the seeding sequence (UGU[G/*A*]CCC) of *miR-938*, which changes *miR-938 *targets. Most interestingly, inflammatory cytokines IL-16 and IL-17A are potential targets of *miR-938 *based on miRNA targetscan analysis (http://www.targetscan.org), and IL-16 and IL-17A may be involved in T1D development [5,6]. Thus, the mutation in the *miR-938 *seed sequence makes *miR-938 *unable to suppress IL-16 and IL-17 expression and may enhance IL-16 and IL-17 production in T1D patients.

IDDM10 is located in a 23Mb region on chromosome 10p12-q11, and the susceptibility gene for IDDM10 is still currently unclear [7]. The potentially functional relevance of *miR-938 *makes the *miR-938 *SNP a very attractive candidate mutation in IDDM10. We hypothesized that *miR-938 *may be a susceptibility gene for IDDM10. In an attempt to test this, we assessed the possible association of the *miR-938 *SNP with T1D in an American Caucasian population. The study population consists of T1D patients and healthy controls from Georgia. The data set includes 622 patients and 723 controls. All diabetic patients were diagnosed using the criteria of the American Diabetes Association. These patients and control subjects have been used in the previous genetic studies [8]. The SNP was genotyped using the TaqMan-assay method [8]. The assay (primers and probe) used in this study was designed and validated by Applied Biosystems. Amplification reactions were performed in a 5ul final volume in optical 384-well plates. PCR was carried out with 2 min at 50°C, 10 min at 95°C followed by 40 cycles of 15 s at 95°C and 1 min at 60°C using an ABI9700 real-time PCR system (Applied Biosystems). The genotypic frequencies for the *miR-938 *SNP are presented in Table 1. The control and patient populations were found to be in Hardy-Weinberg equilibrium (P > 0.05). The frequencies for the *miR-938 *genotypes and alleles (Table 1) were similar between T1D patients and control subjects (P > 0.05). Our data from our Georgia sample, therefore, do not support the association between *miR-938 *and type 1 diabetes. Recently accumulated genetic studies suggest that genetic heterogeneity or different linkage disequilibrium patterns are responsible for the discrepancies observed in Caucasians T1D. Thus, further studies, including functional relevance of the *miR-938 *SNP and associations observed in case/control studies in other populations, need to be performed.

**Table 1:** Frequency of miR-938 SNP in American Caucasian population

	Diabetic subjects (%)	Control subjects (%)	P value*
**Genotypes**			0.197
G/G	327(52.57%)	385(53.25%)	
G/A	257(41.32%)	277(38.31%)	
A/A	38(6.11%)	61(8.44%)	
**Alleles**			0.663
G	911(73.23%)	1047(72.41%)	
A	333(26.77%)	399(27.59%)	.

*Fisher's exact tests

## Abbreviations

T1D: Type 1 diabetes; miRNAs: microRNAs; SNP: single nucleotide polymorphism.

## Conflict of interests

The authors declare that they have no competing interests.

## Authors' contributions

XM, HH, YXD QSM, and LZ participated in the design of the study and coordination. XM and HH carried out SNP analyses. AML performed statistical analysis. JXS supplied DNA samples. XM, YXD, QSM and LZ drafted the manuscript. All authors read and approved the final manuscript.
